# Enhanced Extraction and Separation with HPLC-DAD of Phenolic and Flavonoid Antioxidants from *Portulaca oleracea* L. Leaves Using Tailored Terpenoid-Based NADES: Comparative Assessment of Antiradical and Antimicrobial Activities

**DOI:** 10.3390/antiox14020132

**Published:** 2025-01-23

**Authors:** Tarik Sebbah, Imene Yahla, Edite Cunha, Ali Riazi, Célia G. Amorim, Joan Manuel Rodriguez-Diaz, Maria C. B. S. M. Montenegro

**Affiliations:** 1 Laboratory of Beneficial Microorganisms, Functional Food and Health, Faculty of Nature and Life Sciences, Abdelhamid Iben Badis University, Mostaganem 27000, Algeria; tariksbh.phd@gmail.com (T.S.); imene.yahla@univ-mosta.dz (I.Y.); ali.riazi@univ-mosta.dz (A.R.); 2 Laboratório Associado para a Química Verde|Associated Laboratory for Green Chemistry (LAQV) of the Network of Chemistry and Technology (REQUIMTE), Department of Chemical Sciences, Faculty of Pharmacy, University of Porto, 4050-313 Porto, Portugal; ecunha@ff.up.pt (E.C.); camorim@ff.up.pt (C.G.A.); 3 Laboratorio de Análisis Químicos y Biotecnológicos, Instituto de Investigación, Universidad Técnica de Manabí, Avenida Urbina y Che Guevara, Portoviejo 130104, Ecuador

**Keywords:** green extraction, environmentally friendly method, antioxidants, *Portulaca oleracea* L. leaves, deep eutectic solvents

## Abstract

This study evaluates Natural Deep Eutectic Solvents (NADES) for extracting antioxidant compounds from *Portulaca oleracea* dried leaves, compared to traditional ethanol extraction. NADES were synthesized using terpenoids (menthol and β-citronellol) and organic acids (lactic and capric acid), characterized by favorable viscosity, density, and pH, ensuring liquid stability at ambient temperature. NADES extraction outperformed ethanol, with NADES 1 yielding the highest bioactive contents: 83.66 Eq GA/mg, 786.55 Eq Q/mg, and 0.78 Eq C/mg versus ethanol’s 58.49 Eq GA/mg, 363.23 Eq Q/mg, and 0.44 Eq C/mg. HPLC-DAD analysis identified higher levels of phenolic acids (caffeic and syringic acid) and flavonoids (rutin and quercetin) in NADES extracts, compounds absent in ethanol. Antioxidant potential, assessed via IC_50_ values, confirmed superior activity for NADES extracts (NADES 1-Ext: IC_50_ 28.10 ± 1.73 µg/µL) compared to ethanol (IC_50_ 1615.97 ± 5.34 µg/µL), and the Trolox method has confirmed extensively this superiority. Additionally, NADES demonstrated improved antimicrobial effects, varying with microorganisms. Despite their high viscosity potentially limiting extraction efficiency, adjusting temperature offers a promising approach to enhance mass transfer. These findings emphasize NADES as a sustainable alternative for bioactive compound extraction, paving the way for optimizing extraction techniques through viscosity reduction strategies.

## 1. Introduction

*Portulaca oleracea* L. (POL), a prominent member of the Portulacaceae family, is renowned for its culinary and medicinal applications. This plant has demonstrated diverse medical uses, showing preventive efficacy against metabolic disorders [1,2,3]. Studies have highlighted its positive effects in reducing blood sugar levels and preventing renal [4], neural [5,6], and cardiovascular complications [6], as well as modulating dyslipidemia [7,8,9]. These therapeutic properties are largely attributed to its anti-inflammatory and antioxidant potential, stemming from its rich composition of bioactive compounds, including phenolic acids (p-coumaric, ferulic, gallic, syringic, and caffeic acids) [10,11], flavonoids, and homoisoflavones [12,13,14].

The global demand for phenolic compounds derived from medicinal plants continues to rise, driven by their applications in health and nutrition. Traditional extraction methods often rely on organic solvents, such as ethanol [15], methanol [16], hexane [17], and petroleum ether [18]. While ethanol and methanol are widely used in phytopharmacy and considered relatively safe, their large-scale use and disposal can pose environmental and health challenges, highlighting the need for more sustainable alternatives for extracting bioactive compounds from plants.

Numerous alternative extraction techniques have been explored, including microwave-assisted extraction (MAE) [19], ultrasound-assisted extraction (UAE) [20], heating and agitation [21], aqueous two-phase systems [22], and ball mill-assisted extraction [23]. While these methods offer notable advantages, such as improved efficiency and reduced solvent consumption, they are not without limitations, including challenges with separation, high costs, and process complexity.

Natural Deep Eutectic Solvents (NADES) have emerged as a promising green alternative, aligning with the principles of green chemistry. These solvents are characterized by their biodegradability and biocompatibility, making them suitable substitutes for conventional organic solvents and ionic liquids [24,25]. NADES not only minimize environmental impact but also enhance the solubility, stability, and bioavailability of active compounds, thereby amplifying their therapeutic potential [26,27,28]. Additionally, NADES have been shown to exhibit antimicrobial [27,29] and anticancer properties [28,30,31], further reinforcing their versatility.

Although numerous studies have demonstrated the efficacy of NADES in extracting bioactive compounds from various plant materials [25], data on their application for phenolic compound extraction from POL leaves remain limited. Recent research by Liu et al. (2022) [32] highlighted the use of hydrophilic NADES for the extraction of dopamine from POL, demonstrating the potential of these solvents for this plant material. Addressing this gap, the present study focuses on the development of an eco-friendly extraction methodology using hydrophobic NADES [33,34] to recover phenolic-rich extracts from POL leaves. The extraction efficiency, total phenolic content, total flavonoid content, and condensed tannins were determined, along with in vitro antioxidant and antimicrobial properties of the resulting extracts. The tailored composition of the NADES used in this study (menthol/lactic acid, menthol/β-citronellol, and β-citronellol/capric acid) was specifically designed to balance hydrophobicity with other interaction mechanisms, enabling the effective recovery of phenolic acids. The determination of bioactive contents was complemented with HPLC-DAD analysis, which separated and identified key antioxidants, including five phenolic acids (gallic, caffeic, syringic, coumaric, and ferulic acids) and two flavonoids (rutin and quercetin). This comprehensive analysis not only highlights the extraction performance of the tailored terpenoid-based hydrophobic NADES but also underscores their ability to selectively extract and stabilize high-value bioactive compounds. Furthermore, the high extraction efficiency observed in our study supports the hypothesis that hydrophobic NADES can act as versatile solvents capable of overcoming traditional solubility constraints. By integrating insights from prior research on hydrophilic NADES and demonstrating the potential of hydrophobic NADES, this work promotes sustainable and environmentally conscious extraction technologies. It contributes to the valorization of high-value natural compounds and enhances their post-extraction stability, underscoring the innovative potential of NADES in the extraction of a broader range of bioactive compounds, regardless of their hydrophilic or hydrophobic nature.

## 2. Materials and Methods

### 2.1. Chemicals and Reagents

All chemicals and solvents were of analytical grade quality or better. For sample preparation, menthol (≥99%, CAS: 89-78-1), β-citronellol (95%, CAS: 106-22-9), lactic acid (85%, CAS: 50-21-5), and capric acid (98%, CAS: 334-48-5) were purchased from Merck KGaA (Darmstadt, Germany). Ethanol (99.9%, CAS: 64-17-5) was acquired from Honeywell Fluka (Seelze, Germany). Folin–Ciocalteu’s reagent (CAS: 9005-32-7), sodium carbonate (99.5%, CAS: 497-19-8), DPPH (≥95%, CAS: 1898-66-4), and Trolox (97%, CAS: 53188-07-1) were obtained from Sigma-Aldrich (Madrid, Spain).

Standards of gallic acid (≥99%, CAS: 149-91-7), L-ascorbic acid (99%, CAS: 50-81-7) caffeic acid (≥98%, CAS: 331-39-5), syringic acid (≥95%, CAS: 530-57-4), coumaric acid (≥98%, CAS: 501-98-4), ferulic acid (≥99%, CAS: 537-98-4), rutin (≥94%, CAS: 207671-50-9), and quercetin (≥95%, CAS: 117-39-5) were purchased from Sigma-Aldrich (Madrid, Spain).

### 2.2. Preparation of Natural Deep Eutectic Solvents (NADES)

The preparation of NADES employed combinations of hydrogen-bond donors and acceptors in molar ratios of 1:1 (Table 1). This was achieved using a heating and magnetic stirring method at 50 °C for 30 to 45 min, as described in previous studies [33,34]. The viscosity of the prepared NADES was measured at 25 °C and 40 °C using an NDJ-8S digital rotational viscometer by Hengping (Shanghai City, China). Additionally, the density and pH were determined at 25 °C using an MDJ-600G densitometer by Xiongfa (Hengshui City, China).

### 2.3. Dried Powder Samples of Portulaca oleracea Leaves

The plant *Portulaca oleracea* L. (POL) was initially identified by Pr. Riazi Ali and sourced from the Mostaganem region in Algeria (35°55.869′ N, 0°5.3508′ E) between May and July 2022. The plant was thoroughly washed, its leaves separated, air-dried in the dark for a week, and then subjected to a 24 h drying in an oven at 40 °C. After drying, the leaves were ground into a powder and subsequently stored at −20 °C.

### 2.4. Preparation of NADES and Ethanolic Extracts

The ethanolic extract was prepared by macerating 10 g of dry *Portulaca oleracea L.* (POL) in 100 mL of absolute ethanol, with magnetic stirring for 24 h in sealed, light-protected bottles. For the eutectic extracts, 10 g of dry matter was added to 100 mL of NADES in opaque test tubes, using the same mass-to-volume ratio. Extraction was carried out with ultrasounds and vortexing at temperatures between 45 °C and 50 °C (±2 °C) for 90 min in cyclic intervals: 30 min of ultrasound-assisted extraction (UAE) followed by 2 to 3 min of vortex stirring. The mixtures were then centrifuged at 500 s^−1^ (30,000 revolutions per minute) for 15 min. The supernatant was collected and stored at −20 °C. UAE was conducted using a sonicator from J-P Selecta (Barcelona, Sapin), with a 5 L capacity, a frequency of 40 kHz, a power of 120 W, and a heating power of 90 W, powered by alternating current (AC) at 220 V/240 V with a frequency of 50/60 Hz.

### 2.5. Spectrophotometric Measurements

Spectrophotometric measurements were conducted using a DLAB SP-UV 1100 spectrophotometer by DLAB Scientific Co. (Shunyi District, Beijing, China). This instrument offers a spectral bandwidth of 2.0 nm, wavelength accuracy of ±0.5 nm, and wavelength repeatability of ≤0.3 nm, with a photometric range spanning −0.3 to 3.0 absorbance units (AU) and 0–200% transmittance (T). The photometric accuracy is ±0.002 AU at 0.5 AU. All samples, blanks, and controls analyzed were fully transparent, indicating complete solubilization of dissolved components without any turbidity, ensuring precise quantification of bioactive molecules and reliable measurements of antioxidant activity.

### 2.6. Bioactive Compounds Assay

#### 2.6.1. Determination of Total Phenolic Content (TPC)

Total polyphenols were determined using the Folin–Ciocalteu (FC) method with some modifications, according to Kupina et al. [35]. In summary, 5 mL of 1:10 diluted Folin–Ciocalteu’s reagent in ethanol was added to 1 mL of diluted sample (at different dilution factors 1/8, 1/16 and 1/20 depending on the different NADES-POL extracts samples) or standard, the different dilution factors were used because the NADES extract was highly concentrated, necessitating these adjustments to ensure accurate measurements of the bioactive compounds. Mix contents were allowed to sit for 4 min. Then, the reaction was neutralized with 4 mL of sodium carbonate solution (75 g/L). The absorbance was measured at 760 nm after 120 min of incubation at room temperature against the blank that was a solution containing 100 µL of the solvent + 500 µL of 1:10 Folin–Ciocalteu reagent + 400 µL of sodium carbonate (75 g/L). Gallic acid was used as the standard to create a calibration curve with concentrations ranging from (16 to 250 μg/mL). The results were expressed in milligrams of gallic acid equivalent (Eq GA) per gram of extract (mg Eq GA/g extract). Although the FC reagent lacks specificity for phenolic compounds, it was used in this study for preliminary qualitative screening. More selective evaluation was performed using chromatographic methods, detailed in Section 3.2.

#### 2.6.2. Determination of Total Flavonoids Content (TFC)

The flavonoid content was determined using quercetin as the standard. A calibration curve was established with concentrations ranging from 0 to 20 µg/mL. For each sample, 1 mL of the diluted extract in ethanol (diluted at various factors: 1/8, 1/16, and 1/20 depending on the concentration of the NADES-POL extract) was mixed with 1 mL of 2% (*w*/*v*) AlCl_3_ ethanolic solution. These dilution factors were used to account for the highly concentrated nature of the NADES-POL extract, ensuring optimal absorbance readings and avoiding saturation of the detector. The mixture was incubated for 10 min in the dark at room temperature, and the absorbance was measured at 430 nm against a blank solution. The blank consisted of 1 mL of the solvent used for dilution and 1 mL of the 2% AlCl_3_ solution. Three replicates were performed for each sample, and the results were expressed as milligrams of quercetin equivalent per gram of extract (mg Eq Q/g extract) [36]. We used the aluminum chloride (AlCl_3_)-based spectrophotometric method (absorbance at 430 nm) for its broad applicability in assessing diverse flavonoids in POL extracts. This was complemented by HPLC for quercetin quantification and specific methods, such as tannin measurement at 550 nm.

#### 2.6.3. Determination of Condensed Tannins (CT)

Following the method described by Ali-Rachedi et al. [37], 15 µL of the extract (NADES-POL extracts, ethanolic extract) or standard were mixed with 1500 µL of vanillin solution in ethanol. Then, 750 µL of concentrated hydrochloric acid (HCl) was added. Absorbance was measured at 550 nm after incubating for 20 min at room temperature. The concentration of condensed tannins was calculated using the regression equation from a calibration curve with catechin (0 to 2000 µg/mL) and is expressed as milligrams of catechin equivalent per gram of extract (mg Eq C/g extract).

### 2.7. Chromatographic Conditions for HPLC-DAD Separation Method

An HPLC-DAD method was developed to analyze polyphenols and flavonoids in the extracts. The analysis was performed on a Jasco LC-4000 system with an AS-4050 autosampler, PU-4180 pump, and MD-4010 diode-array detector. A Thermo Scientific Hypersil Gold C18 column (150 mm × 2.1 mm, 3 μm) served as the stationary phase at room temperature, with detection at 320 nm for phenols. The injection volume was 10 μL. The mobile phases were 0.5% acetic acid in water (solvent A) and pure acetonitrile (solvent B), applied in a gradient: 65% A for 10 min, transitioning to 100% B over 15 min, and then 100% A for 16 min, at a constant flow rate of 1 mL/min. Samples were diluted 1:3 in acetonitrile, mixed, and filtered through a 0.22 μm PTFE filter before analysis.

### 2.8. In Vitro Biological Activities

#### 2.8.1. Antioxidant Activity Assessment with DPPH

The antioxidant activity was evaluated using the [2,2-diphenyl-1-picrylhydrazyl] (DPPH) test. Solutions were prepared as follows: 0.004 g DPPH in 100 mL ethanol, 10 mg extract in 100 mL ethanol/NADES, and 200 µg/mL ascorbic acid in ethanol.

A volume of 2500 μL of DPPH was mixed with 100 μL of either the NADES, NADES-Ext of POL or ascorbic acid (used as a standard) at various concentrations. The mixtures were incubated in the dark at room temperature for 30 min, and absorbance was measured at 517 nm, The control solution used to evaluate antioxidant activity was composed of 100 µL of solvent (ethanol or NADES) + 2500 µL of DPPH at 0.004%, while the blank was the solvent used in the extractions (ethanol or NADES).

The scavenging activity was calculated according to Nasri et al. [38], using the following equation:

Scavenging Activity (%) = (A_0_ − A_1_)/A_0_ × 100

where A_0_ is control absorbance and A_1_ is the absorbance in the presence of the added sample.

The Trolox [6-hydroxy-2,5,7,8-tetramethylchromano-2-carboxylic acid] calibration curve was established by measuring the absorbance of a 100 μM DPPH ethanolic solution after the addition of Trolox at various concentrations (0–40 μM). Absorbance was recorded at 517 nm after a 20 min reaction between Trolox and the DPPH radical, as was described by Lingfeng et al. [39] with some modifications.

The equation for the calibration curve is

Y = −0.0237 x + 1.3513

where Y is the absorbance at 517 nm and x is the Trolox concentration in μM.

#### 2.8.2. Antimicrobial Activity Assessment

This study used 9 microbial strains: 3 Gram-positive: *Staphylococcus aureus* (ATCC 33862), *Bacillus subtilis* (ATCC 6633), and *Bacillus cereus* (ATCC 10876); 5 Gram-negative: *Escherichia coli* (ATCC 25922)*, Salmonella typhi* (ATCC 6539), *Klebsiella pneumoniae* (ATCC 13883), *Proteus mirabilis* (ATCC 12453), and *Pseudomonas aeruginosa* (ATCC 27853); and Candida albicans (ATCC10231). The methodology follows the principles of the disk diffusion method in Mueller–Hinton (MH) agar as described by the European Committee on Antimicrobial Susceptibility Testing [40]. Sterile Wattman paper disks, with a diameter of 6 mm, soaked in tubes containing the sample under testing, were carefully placed on the surface of a suitable agar medium for each strain, such as Milan Hinton medium. This medium was previously inoculated with 100 µL of microbial suspension, with turbidity adjusted to 10^8^ CFU/mL for bacteria and 10^6^ CFU/mL for yeast [41]. Negative controls were used as a reference. Petri dishes were sealed and left to diffuse at room temperature for 1 h, then incubated at 37 °C for 24 h for all microbial agents. Trials were conducted in triplicate. The antimicrobial activity of NADES and POL extracts was determined by measuring the diameter of the inhibition zone around each disk. The size of these zones indicates the effectiveness of NADES and POL extracts on the tested microbial strains.

## 3. Results and Discussion

### 3.1. Determination of Extracted Bioactive Compounds

**Figure 1:**
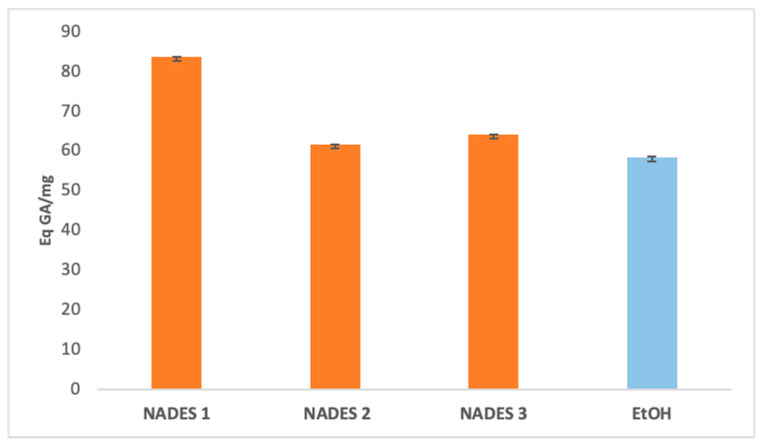
Total phenolic content expressed in (Eq GA/mg) of eutectic extracts and ethanol extract.

**Figure 2:**
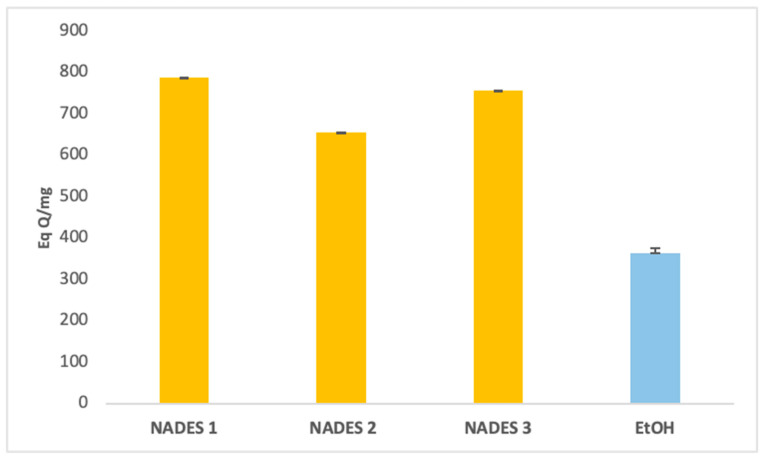
Total flavonoids content expressed in (Eq QA/mg) of eutectic extracts and ethanol extract.

**Figure 3:**
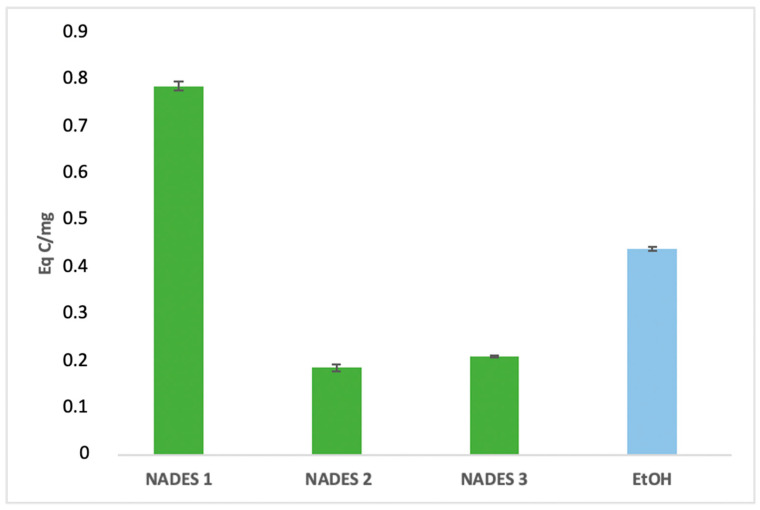
Condensed tannins content expressed in (Eq C/mg) of eutectic extracts and ethanol extract.

The extraction yields of bioactive compounds were significantly higher with NADES compared to ethanolic extracts. Among the tested systems, NADES 1 exhibited the highest yields for all bioactives (83.66 Eq GA/mg, 786.55 Eq Q/mg, 0.78 Eq C/mg) compared to the ethanolic extract (58.49 Eq GA/mg, 363.23 Eq Q/mg, 0.44 Eq C/mg), as shown in Figure 1, Figure 2 and Figure 3.

This superior performance is attributed to the apolar nature of terpenes in NADES, complemented by polar functional groups from organic acids, which offer a broad polarity spectrum ideal for extracting polyphenols, flavonoids, and tannins [42]. Additionally, menthol, a key component in NADES formulations, enhances bioactive yields due to its ability to function as both a hydrogen bond donor (HBD) and acceptor (HBA) [34]. NADES 2 and 3 demonstrated high flavonoid yields (653.31 Eq Q/mg and 754.79 Eq Q/mg, respectively) but showed limited tannin extraction, likely due to their low densities [33,34,42,43,44]. These results corroborate previous studies reporting higher yields when organic-acid-based NADES are used for phenolic [45,46] and flavonoid extraction [47].

### 3.2. HPLC-DAD Separation Analysis

Bioactive molecules were extracted from *Portulaca oleracea* L. (POL) leaves and separated using the validated HPLC-DAD method. The targeted compounds, selected for their potent antioxidant effects, include five phenolic acids (ferulic, gallic, coumaric, caffeic, and syringic acid) and two flavonoids (rutin and quercetin). These compounds are recognized as the primary bioactives contributing to POL’s antioxidant properties. Notably, gallic acid and quercetin were previously utilized as standards for quantifying total phenolic and flavonoid content.

**Figure 4:**
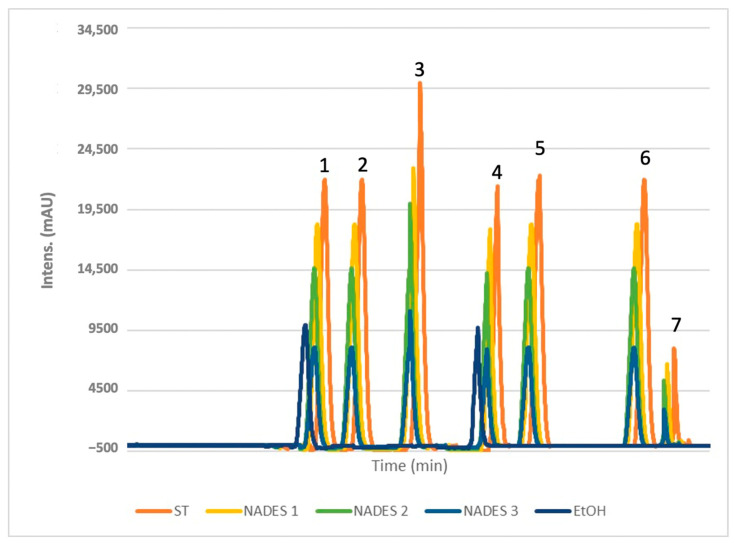
HPLC chromatograms obtained from the analysis of selected phenols (1: gallic acid; 2: caffeic acid; 3: syringic acid; 4: coumaric acid; 5: ferulic acid; 6: rutin; 7: quercetin) and a comparison of four experimental chromatograms showing analyses of POL’s extracts obtained with EtOH and NADES 1-3.

The chromatogram (Figure 4) reveals distinct peaks corresponding to the standards, confirming the presence of the targeted compounds as well as a comparison of four experimental chromatograms showing analyses of POL’s ethanol and eutectic extracts. NADES-based extractions consistently yielded higher concentrations of these bioactives compared to ethanol, demonstrating the superior efficacy of NADES when combined with ultrasound-assisted extraction (UAE). The use of this technique is important because the ultrasounds it generates disrupt the cellulose in the outer cell barrier of the plant matrix, leading to subsequent hydrolysis [48]. Specifically, NADES 2 and NADES 3, characterized by lower viscosities (see Table 1), facilitated higher extraction yields by minimizing molecular resistance and enhancing compound transfer [49]. This effect was particularly pronounced at elevated temperatures, which further reduced viscosity and improved compound mobility and extraction efficiency. These outcomes were unattainable with ethanol. Among the prepared NADES for this study, those with the best extraction yields of phenolics had the lowest viscosity [33,34]. The effect of viscosity was shown by the study conducted by Chan et al. [34], in which outcomes corroborate our data regarding the low viscosity NADES at 40 °C like NADES 2 and NADES 3.

The combination of terpenes and fatty acids in NADES formulations, such as βC/CA in NADES 3, also enhanced extraction yields, likely due to reduced viscosity at 40 °C [50]. For gallic acid, NADES 1 achieved the highest yield (12.7 mg/g), followed by NADES 2 (10.8 mg/g), with NADES 3 yielding 8.0 mg/g. These results suggest that the ratios of terpene to carboxylic acid play a critical role in extraction efficiency.

Caffeic acid exhibited a preference for terpene-based NADES, particularly those with menthol as the hydrogen bond donor (HBD). NADES 2 yielded the highest amount (1.25 mg/g), followed by NADES 1 (1.2 mg/g) and NADES 3 (0.93 mg/g). This suggests that the specific HBD used significantly influences caffeic acid extraction, with reduced affinity observed in acidic environments, as seen in NADES 1.

**Figure 5:**
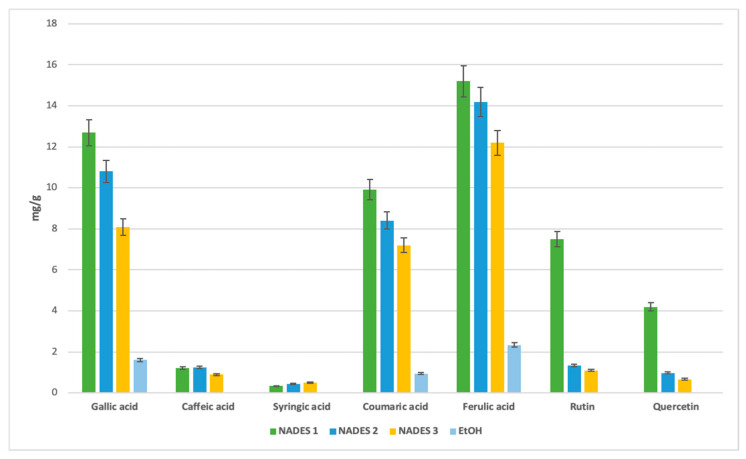
Comparative yield with selected antioxidant compounds of different eutectic and ethanol extracts expressed in (mg/g) separated with HPLC-DAD method.

Certain bioactives, such as syringic, coumaric, and caffeic acids, were undetectable in ethanolic extracts (Figure 5). Syringic acid, while present at lower concentrations across all solvents, exhibited a stronger affinity for NADES.

For flavonoids, the acidic NADES 1 showed the highest yields, with 7.5 mg/g of rutin and 4.2 mg/g of quercetin. These compounds were not detected in ethanolic extracts, further emphasizing the effectiveness of NADES in extracting flavonoids.

#### Method Validation

Method validation is an important part in method development that involves the process of defining analytical requirements and confirming that the method under consideration has performance capabilities consistent with those requirements. The following parameters were evaluated: linearity, precision, detection limit (LOD), and quantification limit (LOQ). The LOD and LOQ were determined at a signal-to-noise ratio of 3 and 10, respectively. Linear calibration curve for the standard was constructed over six calibration levels ranging from 1 µg L^−1^ to 1000 µg L^−1^, each injected in triplicate. The precision was evaluated in terms of repeatability (within-day relative standard deviation, R.S.D.) and in terms of intermediate precision (between-day R.S.D.) at the same concentration level in three non-consecutive days (results displayed in Table 2). The results demonstrated good linearity within the tested concentrations, with determination coefficients (R^2^) above 0.98 for all analytes.

### 3.3. Biological Activities of NADES Extracts

#### 3.3.1. Antioxidant Activity

The antioxidant potential of all extracts was evaluated using the DPPH assay, with IC_50_ values representing the concentration required to achieve 50% radical scavenging activity. These values were compared to that of ascorbic acid, which served as a strong benchmark due to its potent antioxidant properties, reflected in its very low IC_50_ value.

Two primary factors influence the antioxidant effectiveness of the extracts, as highlighted in the literature. First, the extraction yield significantly determines the concentration of antioxidant compounds in *Portulaca oleracea* extracts, directly affecting their antioxidant potential [51,52]. Second, the extractant’s ability to stabilize bioactive molecules depends on various intermolecular interactions within the extract (Figure 1, Figure 2, Figure 3 and Figure 5), as emphasized in previous studies [51,53]. The stabilization of bioactive compounds is further influenced by intrinsic factors, such as the characteristics of the eutectic system components specifically, the hydrogen bond acceptors (HBAs) and donors (HBDs). Extrinsic factors, including extraction time, temperature, and the method employed, also play a significant role in determining overall antioxidant efficacy [54,55].

**Figure 6:**
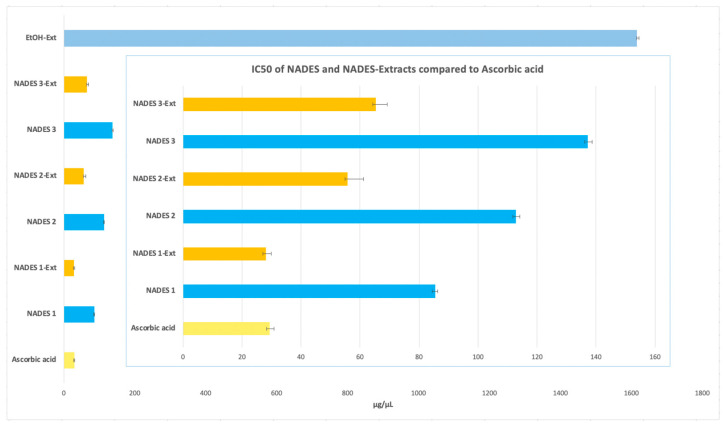
Antioxidant activity expressed with IC_50_ in μg/μL of NADES and NADES containing POL’s extracts.

In this study, all NADES-based extracts demonstrated higher antioxidant potential than the ethanolic extract, which exhibited an IC_50_ value of (1615.97 ± 5.34 µg/µL). Among the eutectic extractions, NADES 1-Ext achieved the lowest IC_50_ value (28.10 ± 1.73 µg/µL), closely matching that of ascorbic acid (IC_50_ 29.39 ± 1.53 µg/µL). NADES 2-Ext and NADES 3-Ext also showed notable antioxidant activity, with IC_50_ values of (55.83 ± 5.35 µg/µL) and (65.31 ± 3.94 µg/µL), respectively, as shown in Figure 6.

When assessing the antioxidant activity of the NADES without the incorporation of the POL’s extract, the IC_50_ values obtained were 85.97 ± 0.97 µg/µL for NADES 1, 113.08 ± 1.30 µg/µL for NADES 2, and 137.43 ± 1.42 µg/µL for NADES 3. These results indicate that the individual NADES systems possess intrinsic antioxidant properties, albeit lower than their corresponding NADES-Ext formulations. This highlights the significant synergistic enhancement provided by the incorporation of bioactive compounds from POL into the NADES systems.

The superior antioxidant performance of NADES 1-Ext can be attributed to the unique property of the eutectic mixture formed by LA and βC as reported by previous studies [56,57]. In addition, an acidic eutectic environment caused by the incorporation or lactic acid (see Table 1) may stabilize antioxidant compounds by inhibiting polyphenol oxidase (PPO), an enzyme responsible for the degradation of phenolic compounds post-extraction [58]. Furthermore, the chelating effect of NADES1 and stabilization of bioactives in NADES1-Ext may also be influenced by the higher viscosity of lactic acid-based NADES, their chelating effects, and their ability to improve the solubility of extracted compounds [54,55].

NADES 2 (M/βC) demonstrated intermediate IC_50_ values, indicating moderate antioxidant activity. This result may stem from the contribution of β-citronellol to the observed activity, although its effectiveness appears limited by the absence of stronger acids, such as lactic acid. On the other hand, NADES 3 (βC/CA) exhibited higher IC_50_ values, suggesting lower antioxidant activity. This could be attributed to capric acid’s greater susceptibility to oxidation compared to lactic acid, despite its previously reported high yields of polyphenols and flavonoids (Figure 1, Figure 2 and Figure 5).

**Figure 7:**
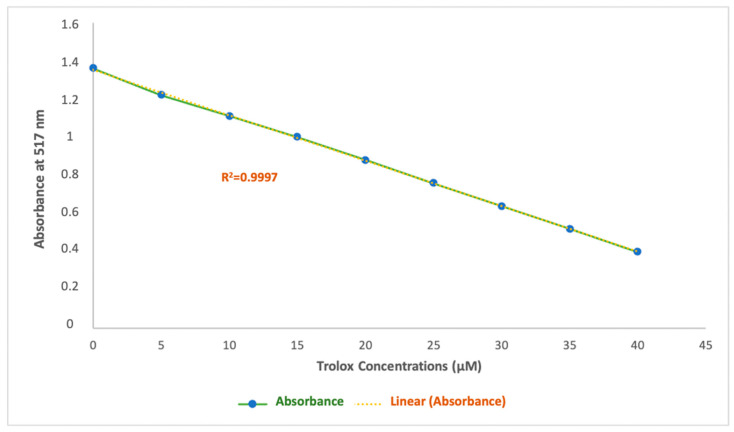
Calibration curve for Trolox, used for DPPH.

A strong linear correlation was observed for the Trolox calibration curve, with a determination coefficient (r = 0.9997) across the concentration range of 0 to 40 μM, with absorbance values ranging from 1.36 to 0.40 nm (Figure 7). The involvement of this extensive method to present the antiradical activity in this study has a major advantage that allows it to be comparable to other studies. Results obtained through this experiment are expressed in Trolox equivalent antioxidant capacity (TEAC_DPPH_) and are shown in Table 3.

The TEAC values obtained confirm the trends previously observed in the antioxidant activity of the samples. Specifically, Trolox equivalents follow a decreasing order among the pure NADES: NADES1 > NADES2 > NADES3, reflecting a gradual decline in intrinsic antioxidant capacity from NADES1 to NADES3 as it was previously discussed through the IC_50_ values. Furthermore, NADES enriched with POL exhibit significantly enhanced antioxidant capacities, following the same trend: NADES1-Ext > NADES2-Ext > NADES3-Ext. These results highlight once again the synergistic effect between NADES and POL bioactive compounds, enhancing their radical scavenging potential.

Conversely, the ethanolic extract (EtOH-Ext) showed extremely low antioxidant capacity, with a TEAC of just 0.11 μM TEq. These findings underline the superior performance of NADES, particularly those enriched with POL, as efficient matrices for the extraction and stabilization of antioxidant compounds.

To summarize, the ethanolic extract of POL displayed the highest IC_50_ value among all tested samples, indicating the lowest antioxidant activity. Conversely, the significantly lower IC_50_ values observed for NADES-based extracts demonstrate their superior ability (to stabilize and enhance the antioxidant properties of bioactive compounds). This study highlights the impact of NADES composition on both extraction yield of antioxidant molecules and their activity. Mechanisms such as increased solubility, metal ion chelation, and the creation of a stable microenvironment contribute to these effects. Notably, the combination of specific NADES components, such as menthol and lactic acid, shows promising potential for antioxidant stabilization. These findings underscore the value of NADES for applications in food, cosmetics, and pharmaceuticals, where maintaining antioxidant stability is crucial. Further research into the specific molecular interactions underlying these systems would be beneficial for optimizing their application and efficacy.

#### 3.3.2. Antimicrobial Activity

The antimicrobial activity of the studied NADES was generally more pronounced against Gram-negative bacteria than Gram-positive bacteria. Among the tested eutectics, NADES 1 demonstrated the most significant inhibitory effects, particularly against Gram-negative bacteria.

The pronounced bactericidal effects of NADES 1 can be attributed to its acidic characteristics, stemming from the presence of carboxylic groups. Although the outer membrane of Gram-negative bacteria typically confers resistance to acidic conditions, the destabilization of inner membrane proteins caused by pH alterations can increase the permeability of the outer membrane. This enhanced permeability creates an inhospitable environment for microorganisms, thereby amplifying the antimicrobial efficacy of the formulations [27,59].

Previous studies have suggested a possible synergistic intramolecular interaction within the NADES structure, involving its hydrogen bond donor (HBD) and hydrogen bond acceptor (HBA) components. NADES systems, particularly acid-based deep eutectic systems, are reported to exhibit greater toxicity than their individual components due to delocalized charges resulting from hydrogen bonding [27,60].

**Figure 8:**
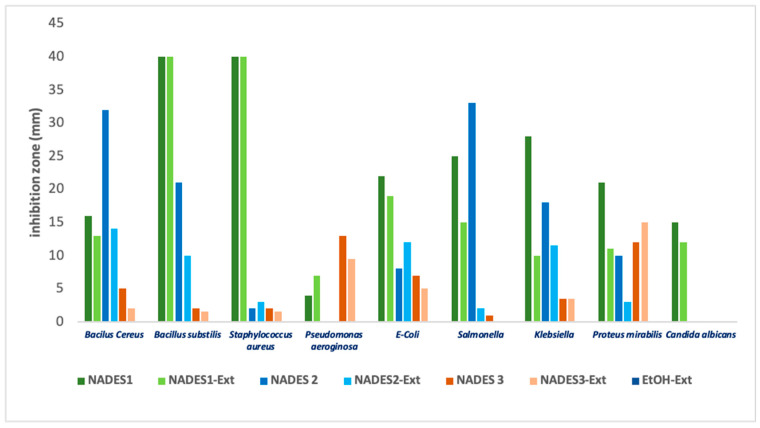
Inhibition diameter (mm) for various observed samples for tested microbial strains.

Among the tested microorganisms, *Staphylococcus aureus* demonstrated the highest sensitivity to NADES formulations containing organic acids (such as lactic acid) and their corresponding POL extracts, as evidenced by the large inhibition zones observed. Conversely, NADES based on terpenes or combined with fatty acids showed reduced efficacy against this pathogen. Interestingly, for NADES 1, the presence of POL’s bioactive compounds negatively affected all tested microorganisms except for *Pseudomonas aeruginosa*. This phenomenon aligns with literature reports indicating that *Pseudomonas aeruginosa* possesses a terpenoid catabolism mechanism [31,61,62]. The limited or negligible inhibition observed for NADES 2 (composed of terpene/terpene) is illustrated in Figure 8 and can be attributed to the inherent resistance of Gram-negative bacteria to natural compounds, including terpenoids [63,64,65,66].

Terpenoids such as β-citronellol and limonene, known for their high solubility and lipophilicity [30,31] significantly reduce the overall viscosity of the eutectic system when combined with capric acid [33], which increases the diffusion coefficient across bacterial cell barriers [31]. However, this increase in diffusion was insufficient to produce a notable inhibitory effect across all tested microorganisms. The effect was more pronounced for *Pseudomonas aeruginosa* when using eutectic mixtures containing fatty acids rather than those with acetic or lactic acids. This finding is consistent with the literature, which notes the variable efficacy of NADES containing organic acids versus fatty acids against Gram-negative bacteria [28,67].

The resistance of Gram-negative bacteria to fatty acid-based antibacterial agents can be explained by their complex cell membrane structure, which includes lipopolysaccharides that repel fatty acids and block their antimicrobial activity [28].

*Candida albicans* showed the lowest sensitivity to all tested NADES and NADES-POL extracts, as indicated by smaller inhibition zones. This reduced sensitivity is consistent with literature reports highlighting the ability of *Candida albicans* to modulate the hydrophobicity of its membrane surface during different growth stages [68,69]. Although medium- and long-chain fatty acids have been documented to exhibit inhibitory effects against *Candida albicans* [69,70], our findings did not show this activity. This lack of effect could be attributed to the reduced efficacy of capric acid when combined with terpenes such as β-citronellol, potentially due to modifications in physico-chemical properties. Eutectic systems are often reported to exhibit novel properties and biological activities that differ from those of their individual components.

In conclusion, these findings underscore the importance of selecting appropriate NADES and plant extract combinations based on the target microorganism. The use of NADES as solvents enhances the extraction efficiency of bioactive compounds from plant materials, thereby improving their antimicrobial efficacy. However, the effectiveness of these formulations varies significantly depending on the type of microorganism being targeted.

## 4. Conclusions

This study explored the potential of NADES for extracting antioxidant compounds from *Portulaca oleracea* dried leaves, comparing their efficiency to traditional ethanolic extraction. Various characterizations, including viscosity and density at 25 °C and 40 °C, as well as pH measurements, demonstrated that NADES, synthesized from terpenoids (menthol and β-citronellol) and organic acids (lactic and capric acid), exhibit a suitable polarity and solubility for bioactive compound extraction. Results showed that NADES, particularly NADES 1, yielded significantly higher amounts of bioactive compounds, including phenolic acids and flavonoids, than ethanolic extracts. The HPLC-DAD analysis confirmed the presence of antioxidant compounds like caffeic acid and syringic acid, which were not extracted by ethanol. Antioxidant activity, as indicated by Trolox equivalent antioxidant capacity along with IC_50_ values, further emphasized the superiority of NADES-Ext, with NADES 1-Ext showing an IC_50_ of 28.10 ± 1.73 µg/µL, outperforming ascorbic acid (IC_50_ 29.39 ± 1.53 µg/µL) and ethanol extract (IC_50_ 1615.97 ± 5.34 µg/µL). Moreover, intrinsic NADES antioxidant properties contributed synergistically to the extract’s efficacy. Antimicrobial activity also benefited from NADES, although its efficacy varied depending on the microorganism. Overall, NADES proved to be more efficient than ethanol for extracting antioxidant compounds, although their high viscosity poses a challenge to extraction efficiency. Reducing viscosity, possibly by increasing extraction temperature, could improve mass transfer and yield. Future studies should focus on optimizing the fluidity, conductivity, and polarity of NADES, as well as assessing their toxicity to ensure their safe application in industrial extractions.

## Figures and Tables

**Table 1:** Physico-chemical comparison of pH, experimental viscosity (μ) values at 25 °C and 40 °C and experimental density (ρ) values at 1 bar and 25 °C between different NADES.

NADES	NADES 1	NADES 2	NADES 3
Composition	Menthol/Lactic acid	Menthol/β-Citronellol	β-Citronellol/Capric acid
Abreviation	M/LA	M/βC	βC/CA
Molar ratio	1:1	1:1	1:1
pH	2.0	3.04	3.13
μ^25°C^ (mPa·s)	75.30	23.30	11.9
μ^40°C^ (mPa·s)	32.30	17.70	10.2
ρ^25°C^ (mPa·s)	0.984	0.880	0.883

**Table 2:** Analytical figures of merit of the method regarding calibration data, precision, and sensitivity.

Analytes	Calibration Equation Y = mX + b	Determination Coefficient (R^2^)	LOD (µg L^−1^)	LOQ (µg L^−1^)	Repeatability (% RSD), *n* = *6*	Intermediate Precision (% RSD), *n* = *6*
Gallic acid	367.3X − 0.07	0.9963	8.9	29.6	2.9	5.2
Caffic acid	97.0X − 0.06	0.9951	9.5	31.6	2.7	5.1
Syringic acid	20.9X − 0.01	0.9956	28.1	93.6	3.3	6.8
Coumaric acid	337.1X − 0.41	0.9986	23.3	77.6	2.5	3.1
Ferulic acid	402.7X − 0.38	0.9922	46.8	155.8	4.7	7.4
Rutin	29.5X − 0.06	0.9870	48.1	160.2	5.8	8.3
Quercetin	133.3X − 0.67	0.9875	39.2	130.5	5.3	7.5

**Table 3:** Trolox equivalent antioxidant capacities (TEAC_DPPH_) of NADES, NADES-enriched extracts, and ethanolic extract expressed in (μM TEq) along with the absorbance of each sample at 50% of its concentration.

Samples	NADES1	NADES1-Ext	NADES2	NADES2-Ext	NADES3	NADES3-Ext	EtOH-Ext
**Absorbance (nm)**	0.324	0.13233	0.579	0.34933	0.71	0.36866	1.325
**TEAC_DPPH_ (μM TEq)**	4.33459916	5.14333333	3.25864979	4.22772152	2.70590717	4.14616034	0.11097046

## Data Availability

Data is contained within the article.
